# Existential Well-Being and Meaning Making in the Context of Primary Brain Tumor: Conceptualization and Implications for Intervention

**DOI:** 10.3389/fonc.2015.00096

**Published:** 2015-04-27

**Authors:** Tamara Ownsworth, Kimberley Nash

**Affiliations:** ^1^Griffith Health Institute, School of Applied Psychology, Behavioural Basis of Health, Griffith University, Brisbane, QLD, Australia

**Keywords:** brain tumor, existential well-being, meaning making, psychosocial support, end-of-life

## Abstract

When faced with a significant threat to life, people tend to reflect more intensely upon existential issues, such as the meaning and purpose of one’s life. Brain tumor poses a serious threat to a person’s life, functioning, and personhood. Although recognized as an important dimension of quality of life, existential well-being is not well understood and reflects an overlooked area of support for people with brain tumor. This perspective article reviews the historical underpinnings of the concept of existential well-being and integrates this discussion with theoretical perspectives and research on meaning making and psychological adjustment to primary brain tumor. We then provide an overview of psychosocial support interventions for people with brain tumor and describe the findings of a recently published psychotherapy trial targeting existential well-being. Overall, this article highlights the importance of assessing the existential support needs of people with primary brain tumor and their family members, and providing different avenues of support to facilitate the meaning-making process across the illness trajectory.

## Introduction

The threat to life associated with brain tumor frequently propels people to consider their own mortality and the meaning and purpose of life (1–3). Spiritual well-being broadly refers to one’s sense of inner peace, connectedness to others, and reverence for life, and encapsulates both religious well-being and existential well-being (2, 4, 5). Spirituality may or may not entail formal religious practices, but relates more generally to people’s propensity to seek meaning in their lives, grow, and transcend beyond the self (4). Existential well-being refers to a person’s present state of subjective well-being across existential domains, such as meaning, purpose, and satisfaction in life, and feelings of comfort regarding death and suffering (6). People’s confrontation or comfortability with such issues signifies their relative levels of existential well-being or distress (7).

## Historical Underpinnings and Conceptualizations of Existential Well-Being

Existential well-being is rooted in the work of existential philosophers such as Kierkegaard and Nietzsche dating back to the nineteenth century (8, 9). *Existentialism* refers to overarching human concepts of personal freedom, suffering and death, and the pursuit of meaning and purpose (9). Existential perspectives focus on the structure of a person’s experience and understanding of self at the level of “being.” The only reality of our existence is what we are conscious of and relate to ourselves in the moment (10). Heidegger (8) and Yalom (9) proposed that threat to life often propels individuals from an inauthentic *business as usual* state where one is unaware of the authorship of one’s life, to a more authentic *mindfulness of being* state where existential themes are considered with greater intensity. Facing mortality may lead to a disequilibrium that provides opportunity for fundamental reconsideration of life values and the meaning of one’s existence (9, 11).

Society’s attitudes toward existential issues underwent great change during the twentieth century (9). Previous generations saw death as a natural part of life, or a cyclic event where generations followed generations and deceased ancestors would greet you after death. Today, many people experience a slower death with terminal symptoms and relatively few people have seen a dead person (12). Death and dying have been transferred from the home to a health care setting where there is usually greater emphasis on physical aspects of quality of life than psychosocial or existential aspects (12–14). Further, a decline in religious beliefs, including the concept of life after death, has not been replaced by philosophical alternatives. Therefore, many believe that death implies annihilation where one ceases to exist, contributing to existential distress (9, 12). Societal changes have meant that existential issues are scantly addressed or discussed in daily life. Consequently, people may not contemplate the meaning of life or death until their own life or a significant other’s life is threatened (14). Death anxiety is common and typically relates to people’s concerns about leaving loved ones, fear of premortal (e.g., pain and suffering) or postmortal (what occurs after death) possibilities, and fear of annihilation (9). Experiencing a threat to one’s life can also propel people to question “have I lived the life I wanted to live?” and motivate them to embrace living (14).

The twenty-first century has seen a greater focus on existential well-being in the context of chronic illness, including conceptualization, measurement, and support needs (2–6, 15–22). Nevertheless, the concepts of spirituality and existential well-being have been referred to as ambiguous and difficult to study (5, 18). Some researchers view spirituality and existential well-being as core dimensions of health-related quality of life that are related to but distinct from physical, emotional, and social well-being domains (6, 18, 22). For example, Brady and colleagues (22) found that spirituality was uniquely associated with quality of life after controlling for physical, social/family, and emotional domains. Others have examined spirituality and existential beliefs as predictors of quality of life and emotional well-being (19, 20). Specifically, existential well-being has been described as an internal coping resource whereby people draw upon their beliefs to cope with stressful situations and improve their emotional well-being (5, 15). Despite different conceptualizations, a consistent finding in the cancer literature is that people with higher levels of existential well-being report lower emotional distress and better quality of life (15, 19, 21, 22).

According to Clarke and colleagues (23), distress in the context of medical illness refers to a range of affective, somatic, dissociative, and grief-related symptoms. They developed an empirically derived taxonomy of common distress syndromes in patients with diverse medical conditions. Differentiated by levels of demoralization, grief, and anhedonia, six main classes were identified, as follows: low distress, uncomplicated grief, moderate distress, anhedonic depression, demoralized grief, and demoralization. The classes with the highest levels of distress (i.e., anxiety and depression) were demoralization and demoralized grief. Demoralization was indicated by a sense of helplessness, hopelessness, and inability to cope, whereas people with demoralized grief additionally recognized a loss of some kind and showed grief-like reactions. Anhedonic depression was characterized by loss of interest and inability to experience pleasure. Importantly, their study indicated that depression with loss of interest and pleasure can occur in the absence of high levels of demoralization or grief (23), Clarke et al. (24) replicated these findings in a severe illness sample including people with metastatic cancers.

Several researchers have assessed both mood symptoms and spirituality and existential concerns in the context of cancer and brain tumor [e.g., Ref. (7, 18, 19, 22)]. Tools assessing spirituality and existential well-being typically include statements regarding feelings and beliefs (7, 18, 22). For example, the Functional Assessment of Chronic Illness Therapy-Spiritual well-being (FACIT-Sp) includes statements such as “I feel peaceful” and “I have a reason for living” [(18), p. 79]. Similarly, the existential well-being subscale of the McGill Quality of Life [MQoL (6)] asks people to rate their feelings and beliefs about life’s purpose and meaning and the future (e.g., not afraid – terrified). Scores on these instruments signify people’s relative levels of existential distress or well-being.

The experience of illness has been found to strengthen some people’s spiritual beliefs or faith (1, 25). Alternatively, for others, the illness may challenge their beliefs about themselves, other people, and the world, and stimulate a search for meaning (25, 26). Existential well-being can be enhanced by *sense making*, or exploring how the event fits with one’s worldviews, and *meaning making* or determining the significance of the event for one’s life (27).

## Meaning Making and Existential Well-Being after Brain Tumor

Primary brain tumor is a unique illness with the combined effects of cancer and brain damage. Therefore, brain tumor poses a threat to a person’s life, functioning, and sense of self (12). People may mourn the loss of changes in their abilities, lifestyle, and years ahead of spending time with one’s family and achieving goals. The impact of brain tumor on existential well-being has mainly been investigated using qualitative methodology [e.g., Ref. (1, 3, 12, 13, 28)]. These particular studies suggest that existential distress is common at different phases of the illness. Adelbratt and Strang (12) found that the possibility of death and an uncertain prognosis propelled some participants to question the meaning and purpose of life. Within these qualitative studies participants expressed fear about separation from family members, a loss of autonomy, and/or anxiety about the unknown. For example, *“I am afraid of vanishing away, and I think of that several times a week”* [(12), p. 503]; *“You sort of look forward and you wonder what’s there”* [(1), p. 131–132].

Another theme emerging from some qualitative studies is the oscillation between hope and despair in the adjustment to brain tumor (12, 13). The struggle with death and dying was implicit from contradictory statements. For example, in the study by Adelbratt and Strang (12), some participants denied being scared by death, but later disclosed that they were afraid of dying and that it was always on their mind. This oscillation was also described by Carvers et al. (13), who found that existential fears were frequently expressed alongside efforts to find meaning in the journey toward death; a state of flux also identified in the broader cancer literature (29, 30).

Existential issues and uncertainty about the future also represent major sources of stress for family members. In qualitative research, some caregivers reported fear and despair concerning their loved ones’ prognosis, and low sense of security in their own lives (31, 32). Some caregivers also expressed that it was difficult to plan ahead due to worry about tumor progression, functional decline, and other set-backs such as seizures (31). In quantitative research, caregivers have been found to endorse high levels of depression (30%) and anxiety [40–60%; (33, 34)], and in one study they reported poorer quality of life than individuals with brain tumor (35).

A literature search conducted on spirituality or existential well-being and primary brain tumor using PubMed and PsycINFO identified two quantitative studies [one full article (7) and one conference abstract (36)]. Pelletier et al. (7) found that up to 50% of their brain tumor sample reported existential distress or death anxiety on the MQoL existential subscale (6). Greater existential distress was associated with poorer quality of life, fatigue, and depressive symptoms. Similarly, Randazzo et al. (36) found a significant positive association between spiritual well-being on the FACIT-Sp12 and health-related quality of life. In our own research (37), we found that levels of existential well-being on the MQoL did not differ according to tumor type, time since diagnosis, or neuropsychological status. However, older age, higher optimism, and lower perceptions of threat and increased perceptions of controllability were associated with greater existential well-being. The relationship between optimism and existential well-being was mediated by perceived controllability, suggesting that optimism is related to better existential well-being through perceptions of controllability. Further, global cognitive status moderated the relationship between optimism and existential well-being, whereby people with high optimism and poor global cognitive function had lower existential well-being than those with high optimism and good global cognitive functioning (37). Higher optimism may promote greater existential well-being by influencing illness appraisals (e.g., increasing focus on aspects that are controllable); however, for people with high optimism, global cognitive impairment may reduce their capacity to maintain a sense of purpose, meaning, and control in life.

The implications of existential fears and concerns for people with brain tumor and their caregivers are vast. Neglect of existential issues is proposed to contribute to despair, loneliness, and anxiety for those with a terminal illness, and may lead to people distancing themselves from loved ones and being distracted from enjoying the pleasures of life (28, 38, 39). Experienced alongside the physical, cognitive, and behavioral effects of brain tumor, existential distress amplifies the negative consequences of a life threatening illness (12, 28).

In contrast, examining the meaning and purpose of one’s life can enhance people’s psychological adjustment to brain tumor (1, 3, 12, 13). Adopting a “sense of coherence” framework, Strang and Strang (3) explored how people make sense of, cope with, and find meaning in their illness. Some participants generated their own theories and explanations for their illness to increase comprehensibility, and drew upon personal and social resources to increase their sense of control and manageability. Other participants expressed that they had strengthened their relationships and redefined life values and roles to find meaning (3). Other qualitative studies have reported similar themes in terms of enhanced relationships, redirecting the focus to living in the “here and now,” and an increased sense of meaning and purpose in life (1, 12, 13). For example, a patient with glioma stated: *“I am looking here and I’m thinking, what are we pushing for all the time? Sometimes you should actually just sit back and enjoy what you’ve got and relax”* [(13), p. 378]. These accounts reinforce existential theorists’ proposition that facing mortality provides an opportunity for reconsideration of life values (9, 10, 14).

Despite the evidence linking existential well-being with lower depression and better quality of life in the brain tumor (7, 36) and broader cancer (15, 19, 21, 22) literature, the supportive care of people with brain tumor often does not reflect this focus. Strang and Strang (28) found that while patients and their caregivers identified existential support as core to holistic care, existential issues were poorly understood by nurses. Nurses reported that patients’ existential concerns were difficult to manage due to time restrictions, their own anxiety, and a lack of knowledge of existential support and related communication skills (3). Similarly, Carvers et al. (13) found that general practitioners’ lack of resources, competency, and communication skills were perceived by patients as barriers to meeting their existential support needs. Communication guidelines for medical practitioners highlight the importance of calming peoples’ fears, discussing the scientific aspects, addressing prognosis issues, forming a partnership with the patient and family, and focusing on their concerns (40). Although these guidelines underscore useful principles for communication, there is a need for support interventions focusing on existential well-being.

## Existential Support Interventions in the Cancer and Brain Tumor Literature

Reviews in the broader psycho-oncology literature have identified various interventions addressing spiritual or existential support needs (41, 42). Henoch and Danielson (41) identified 18 intervention studies for people with cancer, which included hypnosis, individual and group psychotherapy, retreats, psychoeducation, physician counseling, and nurse training. Most of these interventions were multi-faceted with psychoeducation, coping skills, symptom management, and existential support components. A review of 16 positive psychology interventions in breast cancer (42) identified five main approaches, namely, mindfulness-based therapy, expression of positive emotions, spiritual interventions, hope therapy, and meaning-making therapy. Overall, these interventions were found to improve quality of life and different aspects of psychological well-being (e.g., self-esteem, hope, sense of coherence), although methodological quality was variable (41, 42). Examples of controlled interventions focusing primarily on existential issues include cognitive-existential group psychotherapy (43) and meaning-centered group psychotherapy (44). A 20-week group cognitive-existential intervention for women with early-stage breast cancer was associated with significantly reduced anxiety and greater satisfaction with therapy relative to a relaxation only control group (43). The 8-week meaning-centered intervention for people with advanced cancer was associated with significantly greater gains in spiritual well-being and sense of meaning compared to supportive group psychotherapy (44).

A systematic review of supportive care interventions for brain tumor (45) identified mainly case-level descriptions of programs or services providing existential support, such as nurse-led telephone support (46, 47), brain tumor support groups (48), and multi-disciplinary palliative care services (49, 50). Neuro-oncology nursing practitioners have specialized training and expertise in coordinating care throughout the illness trajectory. A vital part of their role entails providing existential support to facilitate adjustment to diagnosis, treatment, and end-of-life issues (51). Our previous research (1) suggested that many people appreciate the opportunity to discuss existential fears and concerns early in the illness rather than support only being offered toward the end of life. This is particularly important given that functional decline associated with a progressive neurological condition can greatly compromise people’s cognitive and communication skills (52).

In contrast to the broader psycho-oncology literature, the main focus of controlled supportive care interventions for brain tumor has been on rehabilitation of physical and cognitive impairments (53). These studies generally support the benefits of rehabilitation for improving cognitive and functional status (53–55); however, gains in mental health and quality of life were not evident after rehabilitation. Furthermore, existential and spiritual dimensions of well-being were not typically focused on or assessed.

## The Making Sense of Brain Tumor Program

To address a major gap in the brain tumor intervention literature, Ownsworth and colleagues (56) developed the “Making Sense of Brain Tumor” (MSoBT) program and evaluated its efficacy in a randomized controlled trial. Conducted in people’s own homes, the 10-session psychotherapy program was guided by the sense of coherence framework (3) and goal-directed. A key focus of the program was on meaning making or supporting people to understand the personal significance of the illness in their own life situation [see Ref. (56)].

Of the 50 people who commenced the program, 44 completed all 10 sessions. After controlling for pre-treatment differences, the MSoBT intervention group reported significantly lower levels of depression, and higher levels of existential well-being (MQOL), functional well-being, and global quality of life at post-assessment than the waitlist group. Significantly lower levels of depression and stress, and higher existential well-being and quality of life were also reported at 6-months follow-up relative to pre-intervention. Importantly, program outcomes did not vary according to tumor type or global neuropsychological status. Having a family member involved in the program was associated with lower levels of depression and better social/family well-being at post-intervention (56). Further, improvement in existential well-being was related to higher levels of therapeutic alliance. These findings highlight that the social context in which people search for meaning and cope in their illness is essential to consider.

## Summary and Future Research Directions

Research indicates that people with brain tumor often experience existential fears and concerns. Unlike the broader cancer literature (41, 42), there are few evidence-based approaches for enhancing existential well-being for this population. The findings of the MSoBT trial (56) indicated that a home-based psychotherapy intervention was effective for improving mood, existential well-being, and quality of life. Providing in-home therapy enabled people with significant physical and cognitive symptoms, and lack of transport, to participate. However, the feasibility and utility of remote access intervention modes (e.g., tele-health) needs to be evaluated. Research by Ownsworth et al. (57) indicated that family caregivers may have both distinct and interrelated support needs. Hence, development of family-system interventions that combine individual, couple, and family-based sessions remain a priority for this population. Furthermore, peer-support interventions may have psychological and social benefits for the neuro-oncology population, such as reducing social isolation, and enhancing morale for the future (48).

Working with people with a terminal illness who experience progressive functional decline can be very challenging, as the topic of death and dying can be an area of disquiet for many health professionals (28). Although professional guidelines for effective communication have been developed (40), further resources and training programs focusing specifically on existential support would be beneficial to enhance the skills and self-efficacy of neuro-oncology practitioners. As highlighted by Strang and Strang (3), most people with brain tumor have spiritual and existential beliefs that support them to find meaning in their illness. Having the opportunity to express one’s fears and values about life and death in a safe and supportive context can make a profound difference to a person’s sense of inner peace and hope for the future.

## Conflict of Interest Statement

The authors declare that the research was conducted in the absence of any commercial or financial relationships that could be construed as a potential conflict of interest.
